# Enamel and dentin in Enamel renal syndrome: A confocal Raman microscopy view

**DOI:** 10.3389/fphys.2022.957110

**Published:** 2022-08-25

**Authors:** Alban Desoutter, Olivier Cases, Pierre Yves Collart Dutilleul, Victor Simancas Escorcia, Vidjea Cannaya, Frédéric Cuisinier, Renata Kozyraki

**Affiliations:** ^1^ Laboratoire Bioingénierie et Nanosciences LBN, Université de Montpellier, Montpellier, France; ^2^ Centre de Recherche des Cordeliers, Sorbonne Université, INSERM, Université de Paris Cité, Laboratory of Oral Molecular Pathophysiology, Paris, France; ^3^ Facultad de Odontología, Universidad de Cartagena, Grupo Interdisciplinario de Investigaciones y Tratamientos Odontológicos Universidad de Cartagena (GITOUC), Cartagena, Colombia; ^4^ CRMR O-RARES, Hôpital Rothshild, UFR d’Odontologie-Garancière, Université de Paris Cité, Paris, France

**Keywords:** confocal Raman microscopy, enamel, dentin, dentin enamel junction, enamel renal syndrome, amelogenesis imperfecta, FAM20A, FAM20C

## Abstract

Enamel Renal Syndrome (ERS) is a rare genetic disorder caused by biallelic mutations in Family with sequence similarity 20A (*FAM20A*) gene encoding the secretory pathway pseudokinase FAM20A. ERS is characterized by hypoplastic amelogenesis imperfecta (AI), impaired tooth eruption, intra-pulpal calcifications, gingival fibromatosis and nephrocalcinosis of various severity. Previous studies showed that the hypoplastic enamel was also hypomineralized but its chemical composition has not been extensively studied. Furthermore it is currently unclear whether dentinal defects are associated with AI in ERS patients. The objective of the study was to provide a structural and chemical analysis of enamel, dentin and dentin enamel junction (DEJ) in ERS patients carrying four, previously reported, distinct mutations in FAM20A. Chemical cartography obtained with Raman microscopy showed that compared to control samples, ERS enamel composition was severely altered and a cementum-like structure was observed in some cases. Chemical composition of peripulpal dentin was also affected and usual gradient of phosphate intensity, shown in DEJ profile, was absent in ERS samples. DEJ and dentinal anomalies were further confirmed by scanning electron microscopy analysis. In conclusion, our study shows that enamel formation is severely compromised in ERS patients and provides evidence that dentinal defects are an additional feature of the ERS dental phenotype.

## 1 Introduction

Dental enamel is the most extreme example of mammalian biomineralization with 96% of the mature tissue occupied by large hydroxyapatite (HAP) crystals organized in 4/5 µm rods. It is the hardest tissue of the human body; its Young modulus ranges from 80 to 100 GPa depending on localization and age (Park et al., 2008). Dentin is also a highly mineralized tissue and HAP crystals constitute 70% of dentin. The dentin enamel junction (DEJ) is a transition zone, including the inner aprismatic enamel and the mantle dentin. Its dimension varies according to localization, type of tooth, but also according to the measurement technique used: from 2 µm with nanoscratching to 11 µm with atomic force microscopy (AFM) (Marshall et al., 2003). Organization of the enamel, dentin and the junction are directly connected to mechanical properties during all life and related to diet of each animal species. Mutations in genes involved in dentinogenesis and amelogenesis perturb this organization and affect the mechanical properties of the dental tissues, essential to mastication and nutrient digestion (Chen et al., 2022).

The Enamel renal syndrome (ERS), a rare defect of biomineralization, is an autosomal recessive disorder characterized by amelogenesis imperfecta, impaired tooth eruption, microdontia, intra-pulpal calcifications and gingival fibromatosis. Nephrocalcinosis of various severity is a classical extra-oral sign of ERS whereas renal cysts, lung and thoracic vertebrae calcifications, hearing loss or intellectual disability are inconsistent findings (MacGibbon, 1972; Jaureguiberry et al., 2012; de la Dure-Molla et al., 2014; Nitayavardhana et al., 2020; Simancas Escorcia et al., 2020; Simancas et al., 2021).

ERS is caused by biallelic mutations in *FAM20A*, a member of the *Family with sequence similarity 20* (*FAM20*) gene family, encoding FAM20A protein. The other two members of the *FAM20* gene family are the secretory pathway kinase FAM20C and the xylosyl-kinase FAM20B involved in proteoglycan biosynthesis (Tagliabracci et al., 2012) (Koike et al., 2009). FAM20C is the Golgi casein kinase phosphorylating serine residues on the SXE/pS motif of more than 100 secreted proteins, i.e., the majority of the secreted phosphoproteome. Several FAM20C substrates including osteopontin, dentin matrix protein or bone sialophosphoprotein are known to regulate bone and tooth development indicating the role of FAM20C in promoting biomineralization (Wang et al., 2010; Ma et al., 2016; Liu et al., 2018). Unlike FAM20C, FAM20A is a pseudokinase. FAM20A forms heterodimers with and allosterically activates FAM20C, *in vitro*. Although still unclear, this process promotes enamel matrix protein phosphorylation and amelogenesis (Cui et al., 2017). Recessive loss-of-function mutations in *FAM20C* result in the Raine syndrome, a rare osteosclerotic bone dysplasia that can be lethal in infancy (Raine et al., 1989; Kingston et al., 1991; Simpson et al., 2007). Raine syndrome is not always lethal and survival into adulthood is possible (Acevedo et al., 2015), (Elalaoui et al., 2016). In these patients the enamel defects, i.e. poorly mineralized, hypoplastic enamel, are reminiscent of the ones observed in ERS subjects (Acevedo et al., 2015). FAM20C dysfunction has also been associated with dentinal defects, i.e., interglobular appearance of the circumpulpal dentin and presence of calcospherites, in patients and mice lacking FAM20C (Acevedo et al., 2015).

Although in rodents FAM20A is expressed in the secretory and maturation stage ameloblasts, odontoblasts and dental pulp cells (O’Sullivan et al., 2011), depletion of murine FAM20A has not been associated with defective dentinogenesis (Li et al., 2019).

Nevertheless, intrapulpal calcifications, a pathognomonic feature of inherited dentin diseases are consistently observed in ERS patients. We indeed previously showed that the ERS pulp stones were composed of carbonated apatite and that their content in fibrodentin and orthodentin was unusually high, possibly suggesting an odontoblast dysfunction (Berès et al., 2018).

Using scanning electron microscopy, energy dispersive spectroscopy and X-ray diffraction we previously showed that ERS enamel was hypoplastic and hypomineralized (Lignon et al., 2017). Considering that enamel formation is controlled by mutual interactions between ameloblasts and odontoblasts, we here sought to assess the structure and chemical composition of enamel, dentin and dentin enamel junction (DEJ) in ERS patients carrying 4, previously published, distinct mutations. c.358C >T (Simancas Escorcia et al., 2020), ERS2: c.1513delA (Jaureguiberry et al., 2012), ERS3: c.907_908delAG, ERS4: c.1432C >T encoding for the proteins p.Gln120*, p.(Ile505Serfs*2), Ser303fsX378 and R478*, respectively.

Here, we combined microcomputed tomography and scanning electron microscopy with high-resolution confocal Raman microscopy to study permanent and deciduous control and ERS teeth. Microcomputed tomography (micro-CT) is a non-destructive technique providing information on tooth mineral density and anatomy. Confocal Raman microscopy is a nonlinear optic technic allowing chemical mapping of dental and other tissues. Raman spectroscopy and microscopy were previously used in dental tissue ultrastructure analysis (Desoutter et al., 2019; Desoutter et al., 2021; Orilisi et al., 2021; Ramakrishnaiah et al., 2015) but not in the context of ERS.

To the best of our knowledge this is the first study using Raman microscopy to determine enamel and dentinal status in ERS patients.

## 2 Material and methods

### 2.1 Ethics—patients recruitment

Patients were referred for oral rehabilitation at the Reference Center of rare dental diseases (Rothschild Hospital, CRMR O-RARE, Paris, France). Diagnosis of ERS was based on clinical and radiological features (Jaureguiberry et al., 2012), (Simancas Escorcia et al., 2020). Patients and healthy age-matched controls were recruited following informed consent in accordance with the principles outlined in the declaration of Helsinki. Written informed consent was obtained from probands for the publication of any potentially identifiable images or data included in this article. The samples used were considered as operating waste according to the French law. Samples from probands and controls were harvested during oral rehabilitation and were prepared for histological analyses (authorization CODECOH DC-2018-3382). The study was approved by the local ethical research committee (process No. 2014-2198).

### 2.2 Samples recovery and preparation

Unerupted and erupted teeth from four ERS patients were included in the study, ERS1/c.358C >T (Simancas Escorcia et al., 2020) (permanent/incisor), ERS2/c.1513delA (Jaureguiberry et al., 2012) (deciduous/molar), ERS3/c.907_908delAG (deciduous/molar) and ERS4/c.1432C >T (deciduous/molar). Teeth removed during oral rehabilitation were washed with distilled water, stored in 0.2% chlorhexidine disinfectant and kept at 4°C until use. Sex and ages at the extraction of patients are as follows; ERS1: male, 26; ESR2: male, 30; ERS3: female, 11; ERS4: female, 15.

After a rough cleaning of the residual tissues around the teeth, sometimes surrounded by calcified tissue, each specimen was cut by an Isomet diamond saw (Isomet 2000; Buehler, Lake Bluff, United States) along the sagittal plane, three or four times to obtain two or three slices, from 400 to 600 μm thick, depending on the size sample; each slice was polished by different discs of different granulometries, 1200, 2400 and on a felt disc by a diamante paste (1/10), and then by solutions containing microstructures of diamonds in suspension (0.05 μm). This step was performed on a Vito TW polishing table (ESCIL^®^, Lyon, France). The samples in the form of longitudinal slices were then cleaned in an ultrasonic tank, twice for 180 s and rinsed with distilled water. To avoid drying out and the appearance of possible fractures, the samples were kept at 4°C with controlled hygrometry.

### 2.3 Histology

#### 2.3.1 Tissue preparation

Teeth were fixed in 4% paraformaldehyde in 0.1 M phosphate buffer (pH 7.4) overnight and then demineralized in buffered 10% EDTA at room temperature under agitation for 4 months. The EDTA solution was changed every week. The sample was rinsed and cryoprotected in 30% sucrose for 1 week. Serial sections of 8 mm thickness were cut on cryostat (Microm). Representative sections were stained with Nissl solution.

#### 2.3.2 Immunocytochemistry

Sections were incubated overnight at 4°C with primary antibodies; mouse anti-Calnexin (1/1000; EPR3632; ab92573; Abcam), mouse anti-CD31/PECAM (1/500; JC/70A; ab9498; Abcam), rabbit anti-FAM20A (1/250; OACD03385; Aviva), and rabbit anti-Fam20C (1/250; OAAB01003; Aviva). Secondary antibodies used were Cy3-conjugated donkey anti-mouse (Thermo Fisher Scientific; 1:500) and Alexa 488-conjugated donkey anti-rabbit (Jackson Immunoresearch Laboratories, West Grove, PA; 1:500). *Helix pomatia agglutinin* Alexa 647 (1/1000; Thermo Fisher Scientific) staining was achieved by overnight incubation. Nuclear staining was achieved by 20 min incubation at room temperature in Hoechst 33342 (Thermo Fisher Scientific). No cellular autofluorescence and no nonspecific labeling were detected in these conditions. Images were collected by confocal microscopy (Zeiss LSM8) and processed using ZEN (Zeiss) and ImageJ softwares.

#### 2.3.3 Scanning electron microscopy

For Scanning electron microscopy (SEM) analysis secondary electron and retro diffused electron images were recorded. SEM was performed with Analytic FEI Quanta FEG 200 microscope (FEI, Hillsboro, OR, United States). Acceleration voltage was 15 kV and pressure was between 10 and 5 Torr. 800 and 12000 times a progressive magnification was used in our experiment. Sample coating was not required for this environmental SEM. SEM analysis allows the visualization of images at high magnification and, hence, it is a useful approach for qualitative analysis of the enamel, dentin and DEJ micromorphology.

#### 2.3.4 Confocal Raman microscopy

The scans were performed on a commercial Witec Confocal Raman Microscope System alpha 300R (Witec Inc., Ulm, Germany). Excitation is assured by a frequency doubled Nd: YAG laser (Newport, Evry, France) at a wavelength of 532 nm. The incident laser beam is focused onto the sample through a ×20 NIKON objective (Nikon, Tokyo, Japan). Resolution of the system is around 300 nm. The scans were performed on an area of around 100 × 100 microns. Each area was scanned by 150 × 150 points, each point corresponding to a spectrum. Area scan was done with an integration time of 0.1 s for sound teeth, and 0.2 s for ERS teeth. Scans were performed for each sample on the amelo-dentinal and cemento-dentinal junctions**.** For ERS samples, scans in amelo-dentinal junctions were performed in areas where pseudo enamel was present. In control samples, equivalent areas were chosen.

Line scans were performed with 2 s integration time, with five spectra recorded and averaged near pulp chamber and near DEJ. Data extraction was performed with the Witec software suite “project five” 5.2, and the processing of the spectra was done with the Spectragryph 1.2.8 software to obtain normalized spectra and further spectra analysis. Raman based images were reconstructed using spectra peak intensity: a peak of interest was selected; for each point of the scanned zone intensity was measured. Using look up tables, each value could be connected to a hue and used to reconstruct a chemical mapping intensity. Phosphate peak intensity and CH peak intensity were reconstructed for each sample. When mentioned, ratios of CH peak intensity to Phosphate intensity were also calculated.

#### 2.3.5 Spectra visualization and normalization

Using the software Spectragryph v1.2.15 spectra were cropped in the region of interest, from 400 to 3,200 cm^−1^. To eliminate background fluorescence, we used the adaptive algorithm, with a coarseness of 15%. Finally, we normalized spectra with the most intense peak (Phosphate PO_4_^3−^) to compare relative intensity. This method allowed to avoid artifacts like difference of intensity due to the laser or the surface of sample.

#### 2.3.6 Microcomputed tomography

To correlate Raman mapping with density of enamel and dentin, high resolution micro tomography was performed. Sound teeth were used as reference of density.

To measure density control and mutant samples were scanned with high resolution computed tomography acquisition and 3D reconstruction using EasyTom 150 RX Solution. To obtain a voxel size of 4.7 * 4.7 * 4.7 mm^3^, voltage source was 87 kV and current 58 µA. An aluminum filter of 1 mm was placed in front of the X-ray source to select hardest beams, reduce intensity and increase contrast of final images. Algorithm transformed radiograph raw images were converted into gray shade TIFF images. Stack of slices were then viewed and analyzed using FIJI v1.53C. To convert gray shades onto HAP density we assumed, as reported by literature, a HAP density of 2.72 g/cm^3^ for sound permanent enamel and 1.97 g/cm^3^ for sound permanent dentin (Gradl et al., 2016), (Schwass et al., 2009) , and 2.81 g/cm^3^ for deciduous sound enamel (Wong et al., 2004)**.** We deduce from this last value a value of deciduous sound dentine for our control of 1.97 g/cm^3^.

### 2.4 Statistics

All statistical tests were performed with SigmaPlot software v11.0. To compare two set of data we used a *t* test, to compare many groups, ANOVA one way test was selected. Each statistical test included a Shapiro-Wilk normality test.

To compare the mineral density within a sample, we used the area selection tool in FIJI software to analyze the regions of interest and values of all the points were collected. For pseudo enamel, the number of points was limited but largely sufficient for parametric statistical tests. A comparison between groups of gray shade values gives the *p*-value two by two.

To evaluate dentinal tubular density, we used several (from five to seven) cutting planes with perpendicular orientation to the tubules. The cutting plane could be different from a slice to another but the space between two tubules, did not depend on the cutting plane. We were therefore able to realize statistical tests.

Finally, we collected Raman spectra in several regions of the dentine, near the DEJ, near the pulp chamber and in intermediate zone and searched for differences in the ratio of CH peak (2,940 cm^−1^) intensity and ν1 of Phosphate (960 cm^−1^).

## 3 Results

The samples used for the analyses are ERS1: c.358C >T (Simancas Escorcia et al., 2020), ERS2: c.1513delA (Jaureguiberry et al., 2012), ERS3: c.907_908delAG, ERS4: c.1432C >T encoding for the proteins p.Gln120*, p.(Ile505Serfs*2), Ser303fsX378 and R478*, respectively.

**FIGURE 1:**
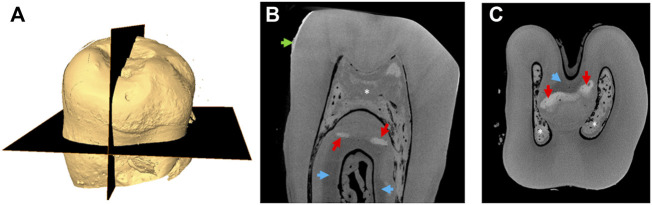
Micro-CT images of an ERS tooth **(A)** 3D reconstruction based on μ-CT imaging; the planes analyzed are shown in black. **(B)** Gray shade micro-CT image from a sagittal section (vertical black plane of A). **(C)** Gray shade micro-CT image from a transverse section (horizontal plane of A). The red arrows indicate zones of increased mineralization. The blue ones indicate hypomineralized zones. The green arrow indicates pseudo enamel layer. Asterisks indicate calcifications in the pulp.

Figures 1A shows a representative 3D reconstruction of an unerupted ERS deciduous molar. The occlusal surface is almost flat due to the strong reduction in the thickness of the enamel layer clearly evidenced in Figures 1B. Although a distinguishable layer of enamel was seen, its mineralization level (enamel grayscale) appeared low in both sagittal and transverse sections (Table 1). The pulp chamber and root canals were filled with a radiopaque calcified material (Figures 1B,C). Areas of altered dentin grayscale indicating zones of hyper- or hypo-mineralization (arrows in Figures 1B,C, Supplementary Figure S1) were also observed and essentially located near the interradicular space.

**TABLE 1:** HAP density of enamel and dentin for each sample, ERS and sound teeth. SD, standard deviation.

	Enamel		Dentin		References
	g/cm3	SD	g/cm3	SD	
a. Sound tooth adult	2.72* ^**,c**^	0.10*	1.97* ^**,c**^	0.04*	Gradl et al. (2016), Schwass et al. (2009)
b. Sound tooth deciduous	2.81* ^**,a,d,e,f**^	0.06*	**1.97** ^**d,e,f**^	**0.04**	Wong et al. (2004)
c. ERS1 (c.358C>T—adult)	**2.46** ^**a,d,e,f**^	**0.06**	**1.91** ^**a,d,e,f**^	**0.04**	
d. ERS4 (c.1432C>T—decid)	**2.25** ^**b,c,e,f**^	**0.07**	**1.98** ^**b,c,e,f**^	**0.03**	
e. ERS2 (c.1513delA—decid)	**2.28** ^**b,c,d,f**^	**0.09**	**2.01** ^**b,c,d,f**^	**0.04**	
f. ERS3 (c.907_908delAG—decid)	**2.13** ^**b,c,d,e**^	**0.07**	**1.91** ^**b,c,d,e**^	**0.03**	

*Values given by literature (see ref).

Small letters under values: Relationship of significant difference.

**FIGURE 2:**
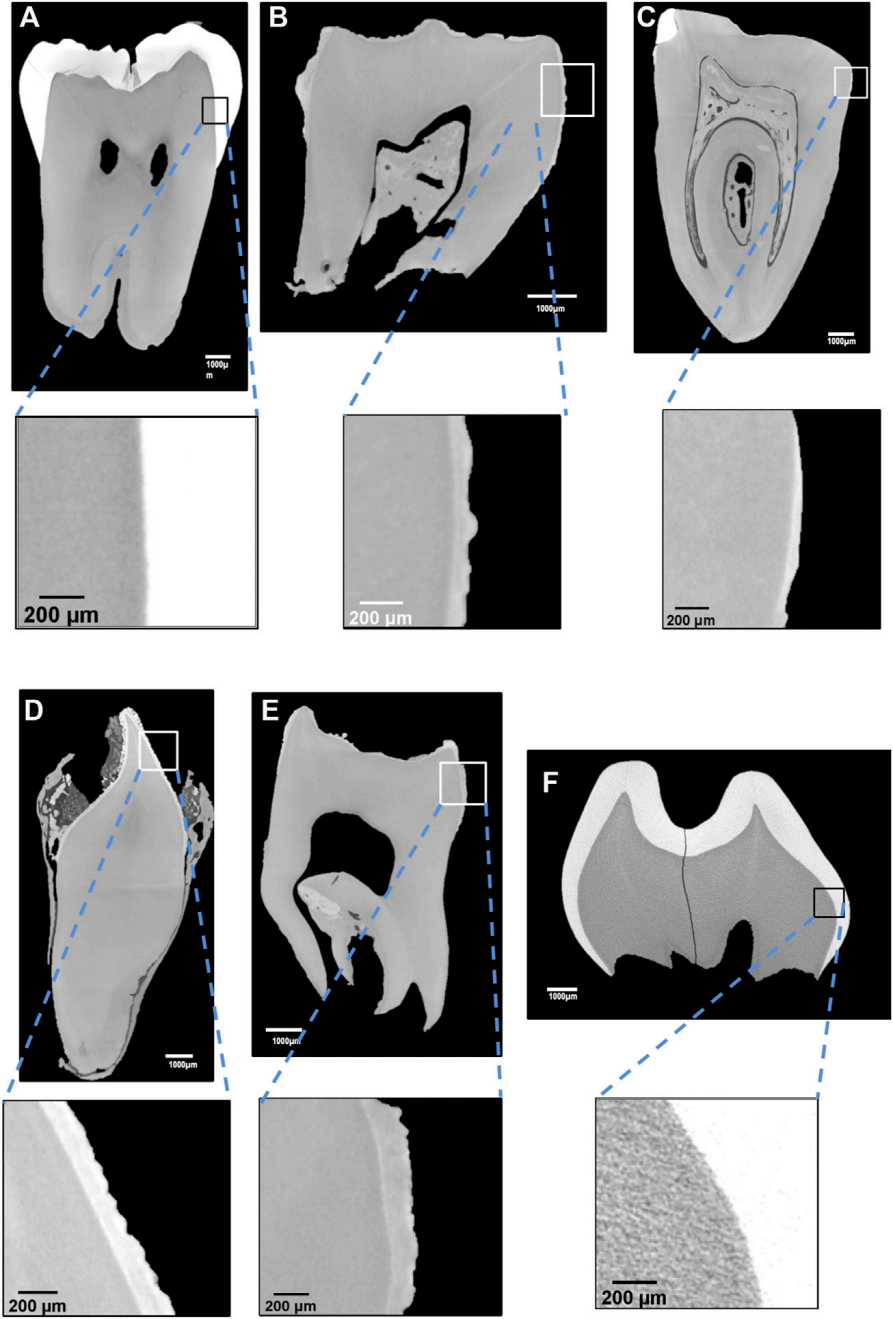
**(A)** Micro tomography gray shade image of DEJ. **(A)** sound permanent tooth; **(B)** ERS3 (deciduous); **(C)** ERS2 (deciduous); **(D)** ERS1 (permanent); **(E)** ERS4 (deciduous); **(F)** sound deciduous tooth; areas of altered gray scale are observed in ERS2 and ERS4.

Micro-CT analysis including in the DEJ zone showed that the affected teeth had an abnormally roughened enamel surface (Figure 2). In the DEJ (boxed areas in Figure 2) the density of the ERS enamel varied between 2.13–2.46 g/cm^3^ and was significantly lower than the one of sound enamel (2.72 g/cm^3^) (Table 1). On the contrary, the density of dentin was lower in ERS1 and ERS3, 1.91 g/cm^3^ vs*.* 1.97 g/cm^3^ of the control sample and higher in ERS4 and ERS2 (1.98 g/cm3and 2.01 g/cm^3^ respectively) (Table 1).

We further analyzed this region using confocal Raman microscopy. Supplementary Figure S2 shows the representative Raman spectra obtained from enamel and dentin for all samples analyzed, including the reference ones. The reference Raman spectrum of enamel was mainly composed of peaks/bands attributed to the mineral apatite (430, 960, 1,072 cm^−1^). Supplementary Table S1. The spectra of ERS enamel displayed a low signal to noise ration. However, unusually large/high peaks in the C-H area (2,940, 2,880 cm^−1^) were observed in ERS3 and ERS4 samples, and to a lesser extent in ERS1 and ERS2, suggesting a previously unreported high organic content. The reference spectrum of dentin indicated the mineral apatite peaks and a higher proportion of C-H stretching bands (2,940 and 2,880 cm^−1^) Supplementary Table S1. This area was modified particularly in ERS1.

Within the DEJ, a structure described as a series of 25–100 μm wide scallops whose convexities are directed towards dentin, the combination of different characteristic peaks of phosphate (960 cm^−1^), carbonate (1,072 cm^−1^) and protein (2,800–3,000 cm^−1^) can provide images of its chemical composition.

**FIGURE 3:**
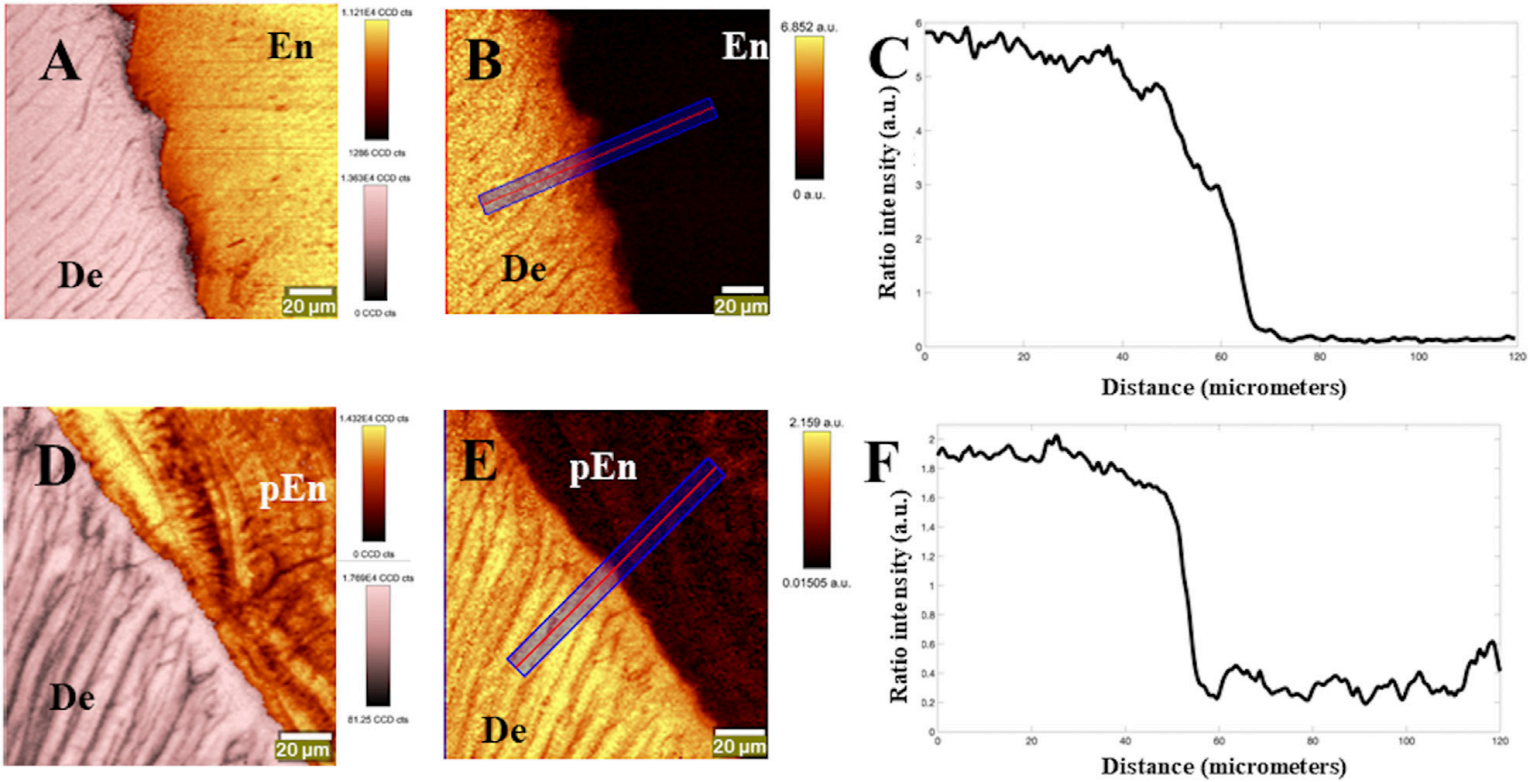
Reconstructed images of control (adult) and ERS1 teeth at the DEJ area. For the conversion in CCDs please use the corresponding lookup tables: **(A–C)** control permanent tooth. **(A)** Chemical combined image of phosphate/960 cm^−1^ peak (yellow hue) intensity and C-H/2,800 cm^−1^ peak (pink hue) intensity at the DEJ; **(B)** Ratio image of CH/2,800 cm^−1^ peak intensity divided by phosphate peak intensity at 960 cm^−1^; **(C)** plotted profile of ratio at the DEJ, along the blue line in **(B)**. **(D–F)** ERS permanent tooth. **(D)** Combined mage of phosphate/960 cm^−1^ peak (yellow hue) and CH/2,800 cm^−1^ peak (pink hue) intensities for ERS1 tooth; **(E)** Ratio image of CH/2,800 cm^−1^ peak intensity divided by phosphate/960 cm^−1^ peak intensity. **(F)** plotted profile of ratio at the DEJ, along the blue line in **(E)**. De, dentin; En, enamel; pEn, pseudo enamel.

**FIGURE 4:**
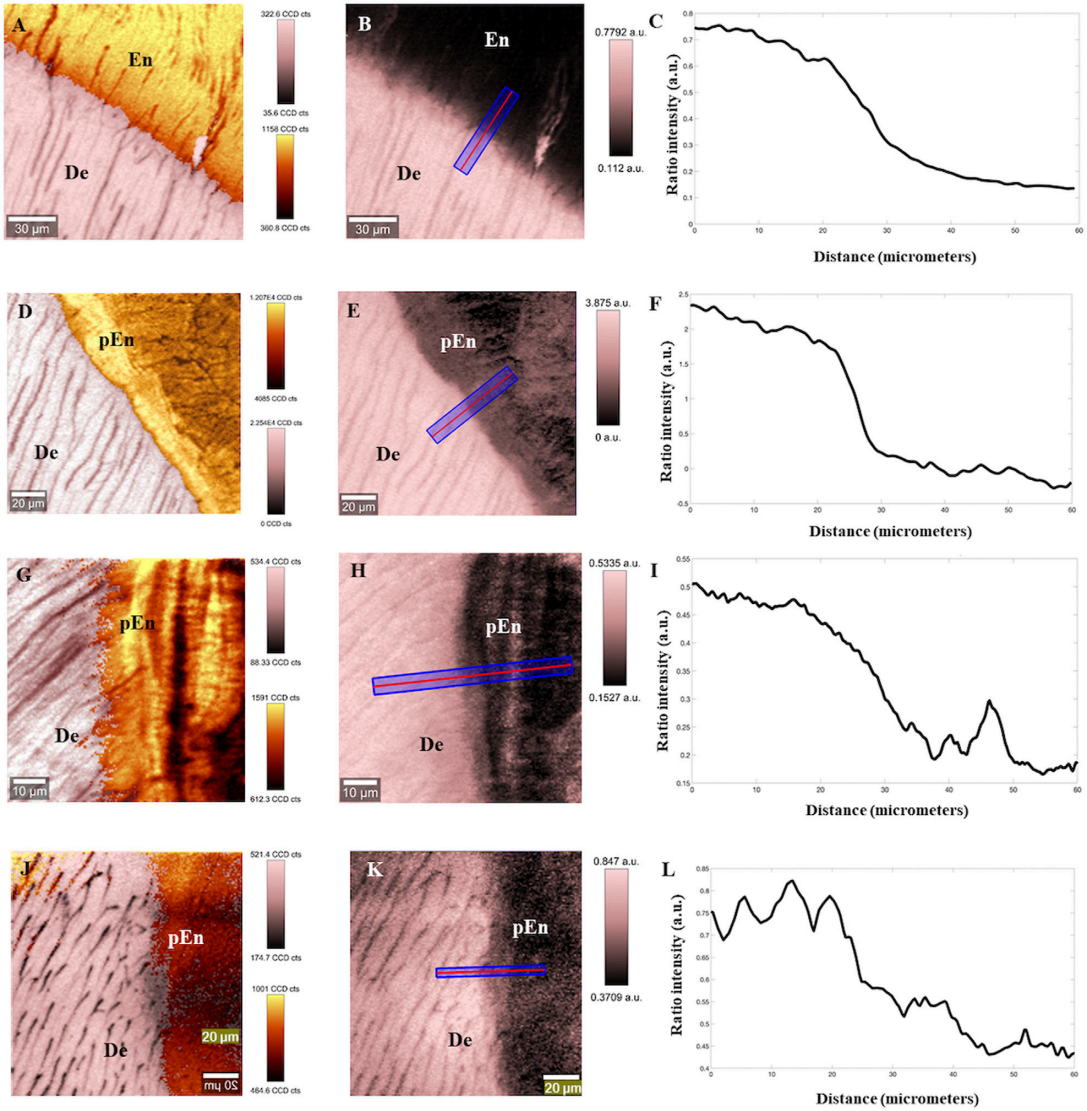
Reconstructed images of control (deciduous) tooth and ERS at the DEJ area. **(A)** Chemical combined image of phosphate/960 cm^−1^ peak (yellow hue) intensity and CH/2,800 cm^−1^ peak (pink hue) intensity of DEJ in sound deciduous teeth with two look up tables; **(B)** image ratio of CH/2,800 cm^−1^ intensity peak divided by phosphate/960 cm^−1^ peak intensity with look up table on sound deciduous sample. **(C)** plotted profile of ratio DEJ, following blue line in **(B,D)** Chemical combined image of phosphate/960 cm^−1^ peak (yellow hue) intensity and CH/2,800 cm^−1^ peak (pink hue) intensity of DEJ in ERS4 adult teeth with two look up tables; **(E)** image ratio of CH/2,800 cm^−1^ intensity peak divided by phosphate/960 cm^−1^ peak intensity with look up table of ERS4 sample. **(F)** plotted profile of ratio DEJ, following blue line in **(E)**. **(G)** Chemical combined image of phosphate/960 cm^−1^ peak (yellow hue) intensity and CH/2,800 cm^−1^ peak (pink hue) intensity of DEJ in ERS2 adult teeth with two look up tables; **(H)** image ratio of CH/2,800 cm^−1^ intensity peak divided by phosphate/960 cm^−1^ peak intensity with look up table of ERS2 sample. **(I)** plotted profile of ratio DEJ, following blue line in **(E)**. **(J)** Chemical combined image of phosphate/960 cm^−1^ peak (yellow hue) intensity and CH/2,800 cm^−1^ peak (pink hue) intensity of DEJ in ERS ERS3 adult teeth with two look up tables; **(K)** image ratio of CH/2,800 cm^−1^ intensity peak divided by phosphate/960 cm^−1^ peak intensity with look up table of ERS3 sample; **(L)** plotted profile of ratio DEJ, following blue line in **(E)**. De, dentin; En, enamel; pEn, pseudo enamel.

Raman based images of the DEJ at the cervical area of control and ERS teeth are shown in Figures 3, 4 for permanent and deciduous teeth respectively.

To enable an accurate determination of sample composition, the images of the spatial distribution of protein (based on intensity of the 2,800 cm^−1^ peak) and inorganic phosphate (based on intensity at 960 cm^−1^) in a control permanent tooth were reconstructed (Figures 3A,B, 4A,B). Depending on the distance from DEJ, the enamel hues varied from bright yellow to orange, the latter corresponding to a lower phosphate content while approaching the DEJ (Figures 3A, 4A). Figures 3B, 4B are Raman images based on ratio between the protein (organic) peak intensity and the phosphate peak intensity. The dark enamel hues reflected the extremely low protein content. Dentin was arranged in parallel tubules with a regular content of phosphate; dentin hues went from orange to bright yellow, the latter corresponding to the high phosphate content of intertubular and/or peritubular dentin. The phosphate and protein intensities could be followed using the lookup tables in Figures 3A,B, 4A,B. The calculated protein to phosphate ratio allowed to distinguish dentin, DEJ and enamel in the area analyzed. The reconstructed Raman image of the permanent ERS1 tooth showed differences in the spatial distribution of both protein and inorganic phosphate (Figures 3D,E, 4D,E,G,H,J,K). The enamel hues were essentially orange throughout the area analyzed indicating a higher organic content and the mutant enamel had a cementum-like appearance with apposed layers (a Raman based image of cementum is shown in Supplementary Figure S3 for comparison) We will refer to this structure as pseudo-enamel (pEn).

Using Raman spectroscopy, a transition zone of about 10–15 mm is classically seen in the normal DEJ (Alebrahim et al., 2015), (Slimani et al., 2017). A characteristic curve of this transition was visible on the graphs accompanying Figures 3C, 4C. In the ERS samples either no transition was seen (Figures 3F, 4F) or the spectra showed a succession of peaks and troughs, corresponding to the different strata of highly or weakly mineralized zones (Figures 4I,L).

A summary of the statistically significant differences in both enamel/pEn and dentin based on peak intensity and gray shade images of control and ERS samples is shown in Table 2.

**TABLE 2:** Statistical difference measurement between sound and ERS sample.

		Sound tooth (deciduous)	Sound tooth (adult)	ERS2 (c.1513delA—decid)	ERS3 (c.907_908delAG—decid)	ERS4 (c.1432C>T—decid)	ERS1 (c.358C>T—adult)
Sound tooth (deciduous)	Raman CH peak	**—**	**		**	*	
	Dentin micro-CT			**###**	**##**	**##**	
	Enamel micro-CT		**§§§**	**§§§**	**§§§**	**§§§**	
Sound tooth (adult)	Raman CH peak		**—**				
	Dentin micro-CT						**##**
	Enamel micro-CT						**§§§**
ERS2 (c.1513delA - decid)	Raman CH peak			**—**	**	**	
	Dentin micro-CT				**###**	**###**	**##**
	Enamel micro-CT				**§§§**	**§§§**	**§§§**
ERS3 (c.907_908delAG - decid)	Raman CH peak				**—**		**
	Dentin micro-CT					**##**	
	Enamel micro-CT					**§§§**	**§§§**
ERS4 (c.1432C>T - decid)	Raman CH peak					**—**	**
	Dentin micro-CT						**##**
	Enamel micro-CT						**§§§**

*Significative difference for 2,942 Raman peak intensity

^#^Significative difference with in gray shade micro-CT image of dentin

^§^Significative difference with gray shade micro-CT image of enamel; number of symbol

^1^ *p* < 0.05; 2: *p* < 0.01; 3: *p* < 0.001

To further investigate dentin composition, we recorded spectra at the DEJ/D-pEnJ (Supplementary Figure S2) and near the pulp chamber (Supplementary Figure S4; Supplementary Table S2). At the D-pEnJ the organic (2,800–3,000 cm^−1^) to mineral (960 cm^−1^) peak ratio was similarly modified in the samples and was consistent with a higher content for groups C-H and CH2 compared to the control. The results obtained near the pulp chamber showed important variability and the dentin organic content seemed to be more important in ERS1, ERS2 and ERS4. The spectra recorded near the pulp chamber of the ERS3 sample could not be exploited due to a too strong presence of fluorescence. A considerable variability was seen for the 2,942 cm^−1^ to 960 cm^−1^ peak ratio: whereas the peak intensity in healthy dentin was 0.36, in the mutant samples the density varied between 0.62 and 0.27. Reconstructed Raman images of the circumpulpal dentin shown in Supplementary Figure S4 further indicated dentinal anomalies particularly evident in ERS3 and ERS1 (Supplementary Figures S4D–F,J–L). The cross sections were plotted and the density of the dentinal tubules statistically evaluated.

**FIGURE 5:**
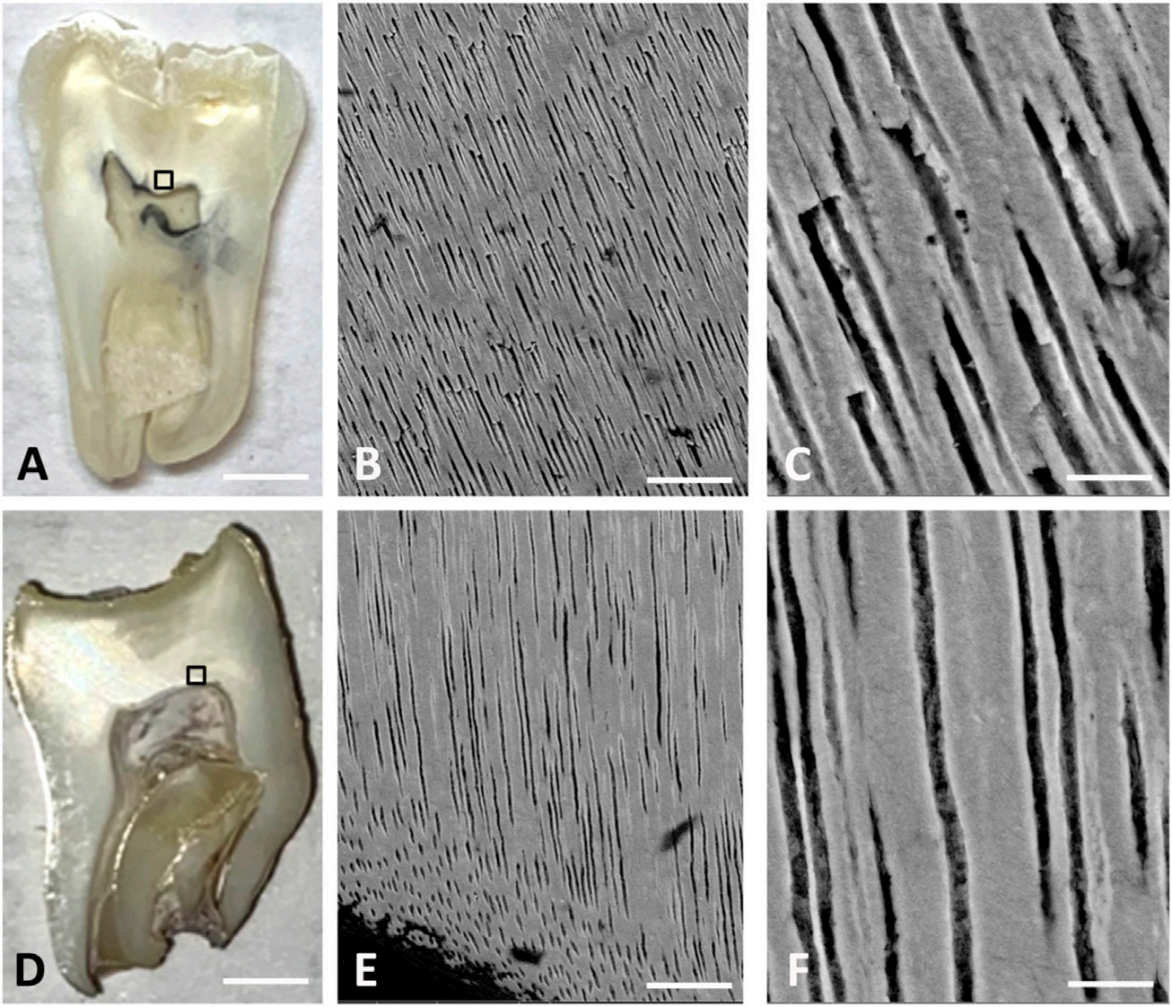
Scanning electronic microscopy. **(A)** Optical image of sound teeth/control; scale bar: 3 mm; **(B)** SEM image of sound dentin; scale bar: 50 μm **(C)** zoom on sound dentin; scale bar: 10 μm; **(D)** ERS4 optical image scale bar: 3 mm; **(E)** SEM image of ERS4 dentin; scale bar = 50 μm; **(F)** zoom on ERS4 dentin; scale bar = 10 μm.

To further investigate dentinal micromorphology we used SEM analysis of dentin near the pulp chamber (Figure 5) and at the DEJ/D-pEnJ area (Supplementary Figure S5).

Ground sections of control and ERS teeth are shown in Figures 5A,D. In addition to the thin enamel layer the roots of the mutant tooth were smaller and misshaped. Pulp chamber and canal calcifications were also evident. Raman spectroscopy analysis confirmed the high mineral content of the pulp chamber (Supplementary Figure S6). The density of this material was very close to dentin and the ratio between the carbonate and phosphate peaks was higher (around 0.2) than the one found in enamel and healthy dentin.

SEM sections showed that the orientation of the tubules in the circumpulpal dentin was not particularly modified although they appeared more loosely arranged than in control teeth (Figures 5B,C,E,F). This observation confirmed Raman microscope scans (Supplementary Figure S3) and showed clear differences in tubular density. Using at least 10 different zones of circumpulpal dentin per tooth, we found that tubular density was statistically modified in all samples analyzed; it was decreased in ERS1 and ERS3, and increased in ERS2 and ERS4.

The dentino-pEnamel junction in ERS teeth exhibited an obvious abnormal flattening and lacked a scallop structure with concaves to the enamel. Consistent with the Raman findings, the adjacent pEn was arranged in a laminar-type pattern (Supplementary Figure S5).

The above observations may suggest that FAM20A is involved in dentinogenesis. We therefore investigated the distribution of FAM20A and FAM20C in human odontoblasts and pulp cells.

**FIGURE 6:**
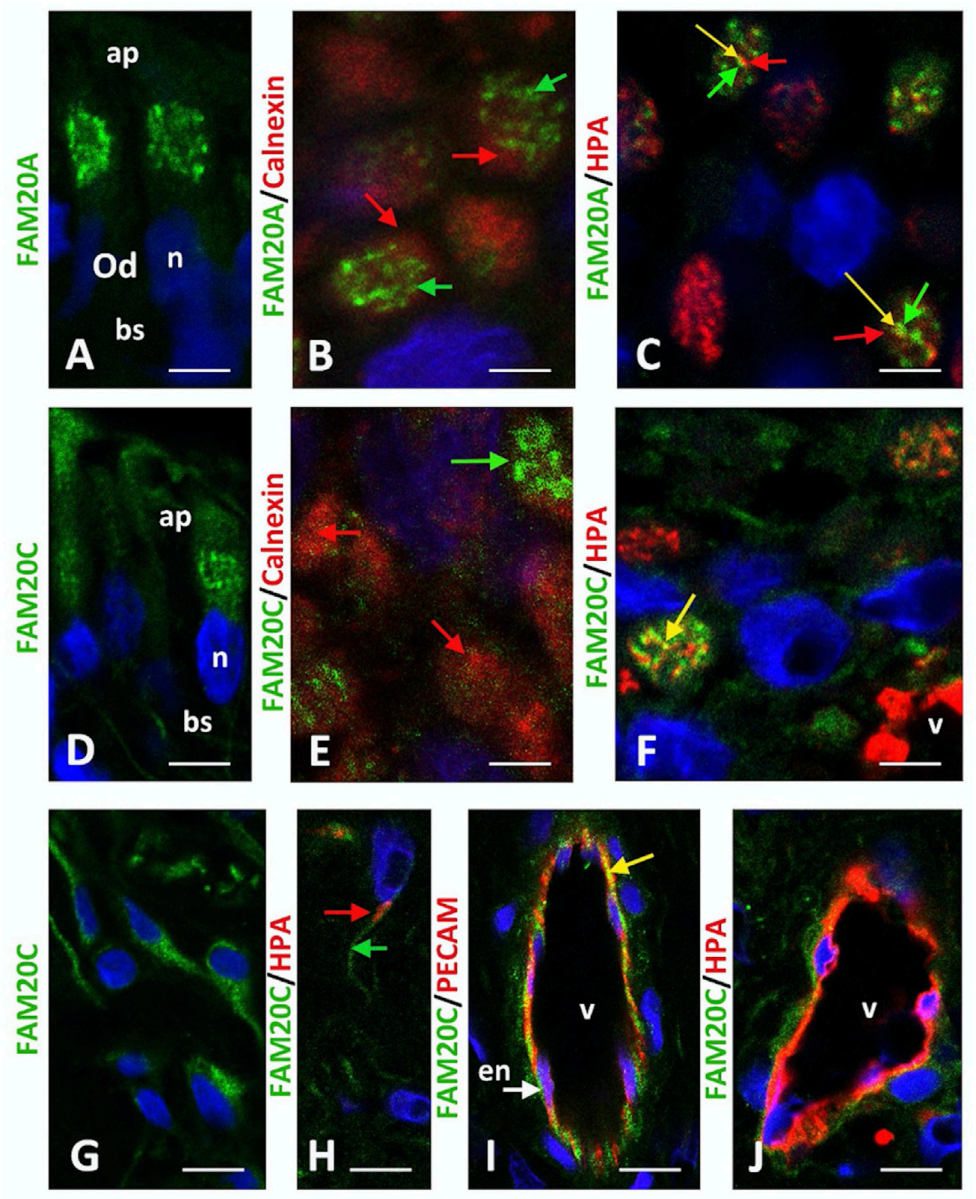
FAM20A and FAM20C immunostaining in permanent teeth. **(A)** In the polarized secretory odontoblast (Od) FAM20A localized to a large structure in the apical domain (ap). **(B)** The merged image indicated that the endoplasmic reticulum marker, Calnexin (red arrows), did not colocalize with FAM20A (green arrows). **(C)** *Helix pomatia agglutinin* (HPA, red), a specific marker of the cis- and medial- Golgi compartments colocalized partly with FAM20A (green) at the interface with the trans-Golgi compartment (yellow arrows). **(D)** FAM20C localized to a large structure in the apical domain of the polarized odontoblast. Scattered vesicles were also found along the cytoplasm and to some extent in the basal domain at the level of the primary cilium (bs, arrow). **(E)** The merged image indicated that the endoplasmic reticulum marker, Calnexin (red arrow), did not colocalize with FAM20C (green arrow). **(F)** HPA and FAM20C colocalized partly at the interface between the medial- and trans-Golgi compartments (yellow arrow). **(G,H)** In pulp fusiform fibroblasts, FAM20C was expressed in small vesicles **(G)** that did not express *HPA* **(H)**. **(I)** FAM20C was expressed in endothelial PECAM-positive cells (yellow arrow) at the interface of the HPA-positive compartment **(J)**. Scale bars: **(A,D,G,H)** = 30 μm. **(B,C,E,F)** = 10 μm. **(I,J)** = 60 μm. en, endothelial cell; n, nucleus.

Using a permanent maxillary molar, we found that FAM20A and FAM20C were strongly expressed in human odontoblasts (Figure 6, Supplementary Figure S7 for a low magnification image of the region analyzed).

To define FAM20A and FAM20C distribution at the cellular level, we used co-staining with calnexin, a typical endoplasmic reticulum (ER) marker, and *Helix Pomatia Agglutinin* (HPA), a marker of the cis- and medial Golgi compartments. Neither FAM20A nor FAM20C staining was detectable in the ER (Figures 6B,E). Both proteins co-localized with HPA, albeit partially (Figures 6C,F). FAM20C was also readily detected in pulp fibroblasts and endothelial cells, also stained by PECAM (Figures 6G,H,I,J). We were unable to detect FAM20A in these cell types.

## 4 Discussion

We here combined various morphological and imaging approaches, including Raman microscopy, to analyze the microstructure, density and chemical composition of enamel and dentin from ERS patients and control subjects. We, in the present work, showed a previously unreported variability in ERS enamel organic and mineral composition and provided clear evidence that the mineralization level, organic content and structure of ERS dentin were also modified, albeit to a lesser extent compared to the enamel. We finally showed for the first time that FAM20A and FAM20C were strongly and closely expressed in young human secretory odontoblasts suggesting that the impaired FAM20A–FAM20C interaction in this cell type could contribute to the dentinal defects.

Micro-CT analysis revealed an important heterogeneity in the mineral density of ERS dental tissues. Enamel formation appeared severely affected in all ERS samples with a thin layer of poorly mineralized enamel, clinically manifesting as hypoplastic (Lignon et al., 2017), an observation consistent with previous reports (Jaureguiberry et al., 2012), (Simancas Escorcia et al., 2020), (Martelli-Júnior et al., 2008).

At the dentinal-pEn junction area the density of the pEn was consistently low with recorded values 10%–22% lower to the control ones. Dentin density was also modified but depending on the sample/mutation analyzed, it was either lower or slightly increased. Despite the observed intervariability, the density values of enamel and dentin appeared to be correlated in the four samples examined.

To obtain a high-resolution chemical and morphological map, we further analyzed the DEJ area of ERS teeth using confocal Raman microscopy. Using samples from four unrelated patients/mutations and an optical resolution of 300 nm we were able for the first time to explore in parallel enamel and dentin in the DEJ area or near the pulp chamber (summary table). Our results revealed that the chemical signature; i.e., nature of the recorded spectra, as well as the laminar-type pattern of the ERS pEn were closer to cementum, a pattern also confirmed by our MEB data and published results (Histology of human cementum, 2016)**.**

We also showed that the phosphate gradient in the junction between pEn and dentin was either absent or abnormally formed. These observations confirm and further the previous published data obtained from one ERS patient (Lignon et al., 2017); they moreover show that the organic content is increased in the hypomineralized ERS enamel. Based on our results we propose that the ratio of peak intensities 2,800–3,000 cm^−1^ to 960 cm^−1^ could be a biomarker of ERS, or at least a specificity of some mutants. The high Raman stretching peaks at 2,942 cm^−1^ may also indicate the presence of phospholipids.

A high organic content perturbs the process of amelogenesis and may result to loosely connected HAP crystals and enamel hypomaturation in addition to hypomineralization. This observation explains the clinical findings of ERS teeth as well as the currently limited restorative and orthodontic options. Indeed, a full-crown treatment is usually proposed as an optimal strategy.

The morphology of the DEJ was profoundly perturbed. The width and scalloping of the mutant dentin-pEn junction were decreased and the organic to mineral peak ratio indicated a high content for protein groups. Based on our results we propose that the ratio of peak intensities 2,800–3,000 cm^−1^ to 960 cm^−1^ could be a biomarker of ERS, or at least a specificity of some mutants. The high Raman stretching peaks at 2,942 cm^−1^ may also indicate the presence of phospholipids.

With respect to the orientation and distribution of the dentinal tubules SEM analysis, micro-CT and Raman microscopy confirmed the enamel and dentin defects previously mentioned in two different cases of ERS patients (Martelli-Júnior et al., 2008), (Dourado et al., 2019). Our data furthered these observations and showed that dentinal tubule density was statistically modified in all ERS samples analyzed, with the most significant difference observed in the ERS3 sample. However, both increased and decreased dentinal tubule density was found. The reason for this variability is currently unknown.

It is interesting to note that dentinal anomalies were previously described in Raine Syndrome, caused by homozygous FAM20C mutations. In these patients however the dentinal phenotype appeared more severe and could at least partially be explained by systemic hypophosphatemia, a prominent feature of RS. Supporting this hypothesis, a high phosphate diet improved the dentin volume and mineralization in Fam20c null mice (Zhang et al., 2021).

None of the patients reported here had overt hypophosphatemia, or abnormal circulating vitamin D or PTH levels. It is thus possible that FAM20A together with FAM20C are locally required for optimal odontoblast secretory function and dentin mineralization. To our surprise and despite an extensive pulpal and radicular calcification in ERS teeth, no FAM20A was found in control pulp fibroblasts or endothelial cells. Further investigations are required to characterize the pathogenesis of the ectopic calcifications. We anticipate however that aberrantly expressed and/or mislocalized mutant forms of FAM20A (Simancas et al., 2021) may contribute to the process by favoring abnormal FAM20C activity.

Our data reveal a variability in the dental phenotype. The four probands carry homozygous mutations which are either nonsense (ERS1, ERS4) or frameshift (ERS2, ERS3) invariably resulting in FAM20A truncation. We previously showed that ERS1 is a null mutation (Simancas Escorcia et al., 2020); ERS2 (I505Sfs*) and ERS4 (R478*) occur in exon 11 in the C-terminal domain of FAM20A, whereas ERS3 (S303fs*) modifies exon six within the N-terminal region of the protein.

It is now established that FAM20A is a pseudokinase with the kinase core extending from amino acids 160–525 (Cui et al., 2017). Mutations in residues K129, K233, S346, and M235 result in ERS and were shown to be important for ATP binding to FAM20A. The role of the mutations reported here is not established but may not impact ATP binding or FAM20A homodimerization shown to include residues 294–254 and 353–358. However, S303 is close to residues involved in FAM20A/FAM20C heterodimerization. Because heterodimerization is sufficient to allosterically increase FAM20C activity (Cui et al., 2017), one may assume that this mutation could decrease FAM20C kinase activity in the mutant tissues.

Not surprisingly the ERS1 “null” mutation resulted in a severe dental phenotype. It is currently unclear how the R478* mutation may affect FAM20A functions and why it profoundly modifies amelogenesis. One hypothesis is that the pseudokinase activity involving residues D430 and D458 of FAM20A may somehow be altered. Alternatively, impaired activation of the TGF beta signaling pathway (Simancas et al., 2021) essential for ameloblast function (Gao et al., 2009) may contribute to amelogenesis imperfecta.

The limitation of the present study is the small number of samples, which is however specific to the rarity of the disease. A better knowledge of FAM20A activities and a larger number of samples, particularly permanent teeth from additional ERS patients would allow to provide genotypic-phenotypic correlations with respect to the enamel and dentinal defects.

Our results are consistent with previous studies and confirm defective enamel formation and dentinal defects of variable severity. The role of FAM20A on odontoblast function requires further investigation and Raman microscopy-based approaches on a larger number of samples will be very informative. Providing fundamental evidence on enamel and dental defects is essential to choose an optimal therapeutic strategy and provide clues for dental tissue engineering.

## Data Availability

The raw data supporting the conclusion of this article will be made available by the authors, without undue reservation.

## Supplementary material

SUPPLEMENTARY FIGURE S1 Mineral defect in ERS dentine samples. **(A)** micro CT gray shade image of ERS2 dentine; **(B)** plotted profile of yellow line seen in **(A)**; **(C)** micro CT gray shade image of ERS4 dentine; **(B)** plotted profile of yellow line seen in **(D)**.

SUPPLEMENTARY FIGURE S2 Normalized Raman spectra of sound (permanent and deciduous) and ERS enamel **(B)** and dentin **(B)**. ERS1: c.358C >T, permanent; ERS2: c.1513delA, deciduous; ERS3: c.907_908delAG, deciduous; ERS4: c.1432C >T, deciduous.

SUPPLEMENTAL FIGURE S3 Example of permanent teeth cementum scanned with confocal Raman microscopy **(A–C) **compare with ERS4 **(D–F)**. **(A,D)**: ×20 optical image; **(B,E)**: 960 cm^−1^phosphate peak intensity reconstructed image; **(C,F)**: 2,800 cm^-1^ CH peak intensity reconstructed image; next to Raman image: look up table and plotted profile of intensity, linked with blue line. **(G)**: superposed non background substracted Raman normalized spectra of cementum of sound tooth and pseudo enamel found in ERS4. Spectra representative of the two samples.

SUPPLEMENTARY FIGURE S4 Dentin structure of ERS teeth scanned with confocal Raman microscope. Left: ×20 optical image. Middle: 2800 cm^−1^/CH peak intensity image. Right: 70 μm intensity profile along blue line on CH peak intensity image. **(A–C)** sound adult dentin; **(D–F)** ERS1; **(G–I)** sound deciduous dentin; **(J–L)** ERS2; **(M–O)** ERS3; **(P–R)** ERS4; Statistical differences in dentinal tubule density are shown (*): p<0.05; (**): p<0.01; (***): p<0.001.

SUPPLEMENTARY FIGURE S5 Scanning electron microscope image of dentin—pseudo enamel interface. **(A) **ERS2; **(B)** ERS3. De, dentine; PEn, pseudo enamel.

SUPPLEMENTARY FIGURE S6
**(A,B)**: classical microscopy image of ERS2 crown and root, ×2.5 magnification; **(C)** single spectra of material in the pulp chamber seen on **(A,B)** with 2 s integration time; **(D)** optical ×20 magnification pulp chamber of ERS2; **(E)** 960 cm^−1^ photosphate peak intensity image of the zone boxed in **(D)**; **(F)** 2,800 cm^−1^ CH peak intensity image of the zone boxed in **(D)**.

SUPPLEMENTARY FIGURE S7 FAM20A and FAM20C immunostaining. **(A)** Low power magnification of a section at the level of the coronary pulp stained with the vital marker, Nissl. Arrow indicated the odontoblast layer (Od). Low power magnifications indicating Fam20A **(B)** or FAM20C **(C)** expression within odontoblast layer (Od). V, vessel. Scale bars: **(A)** = 250 μm; **(B,C)** = 50 μm.
