# Supplementary material for: Enamel and dentin in Enamel renal syndrome: A confocal Raman microscopy view
Source: Front Physiol. 2022 Aug 25;13:957110. doi: 10.3389/fphys.2022.957110 (PMC9453029; doi:10.3389/fphys.2022.957110)
Supplement: Supplementary file 1 [file Table1.docx]

| Raman band assignments | Enamel | Dentin |
| --- | --- | --- |
|  | (Raman shift in cm^-1^) | |
| ν_2_ Phosphate (PO_4_^3-^) | 433-449 | 432-449 |
| ν_4_ Phosphate (PO_4_^3-^) | 579-608 | 580-610 |
| ν_1_ Phosphate (PO_4_^3-^) | 960 | 960 |
| Bending mode Carbonate (CO_3_^2−^)  Stretching mode Carbonate (CO_3_^2−^) | 1044  1070 | 1048  1074 |
| (NH) Amide III |  | 1243 |
| (NH) Amide III non-polar triple helix of collagen |  | 1275 |
| CH_2_ wagging |  | 1450 |
| Advanced glycation end products (AGEs)-Pentosidine |  | 1550 |
| Amide I (C=O) |  | 1660 |
| CH |  | 2800-3000 |
| OH | 3580 | 3580 |
| Phosphate to organic matrix ratios | 960/1450 | 960/1450 |

**Supplementary Table 1**. Characteristic Raman peaks of enamel and dentin
