# Supplementary material for: Enamel and dentin in Enamel renal syndrome: A confocal Raman microscopy view
Source: Front Physiol. 2022 Aug 25;13:957110. doi: 10.3389/fphys.2022.957110 (PMC9453029; doi:10.3389/fphys.2022.957110)
Supplement: Supplementary file 2 [file Presentation1.pdf]

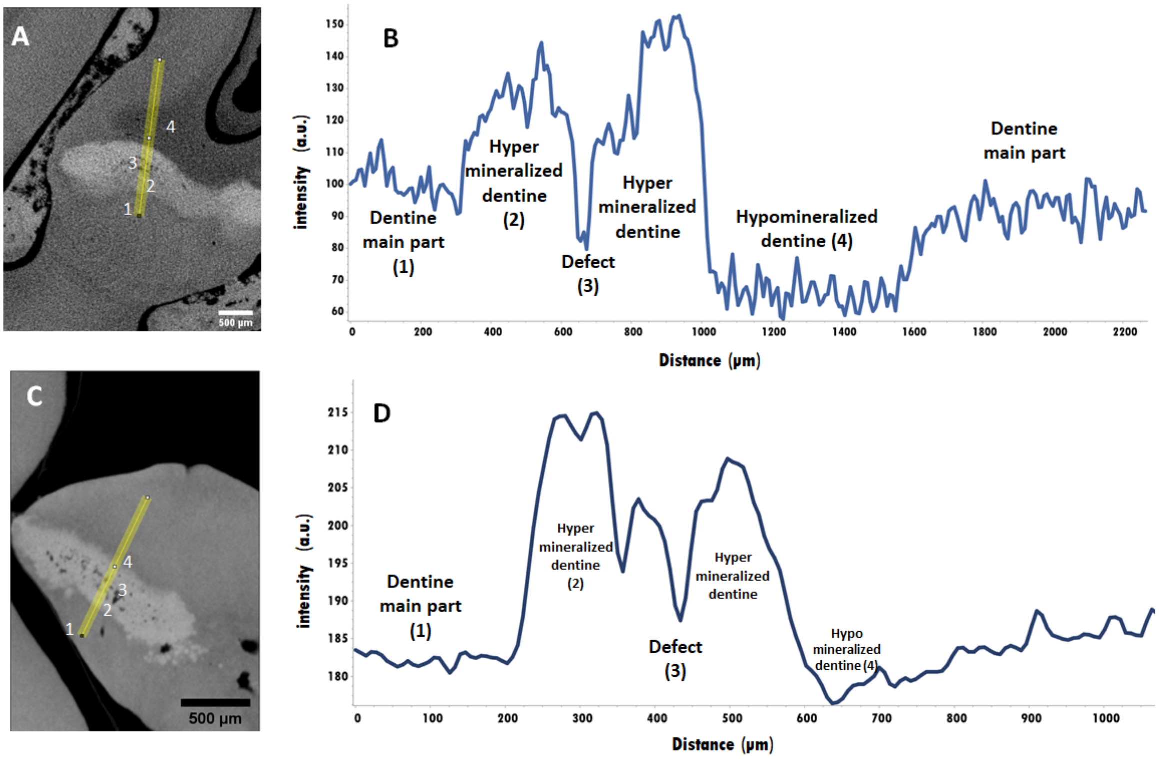

Supplemental figure 1. Mineral defect in ERS dentine samples. A) micro CT gray shade image of ERS2 dentine; B) plotted profile of yellow line seen in A; C) micro CT gray shade image of ERS4 dentine ; B) plotted profile of yellow line seen in D;

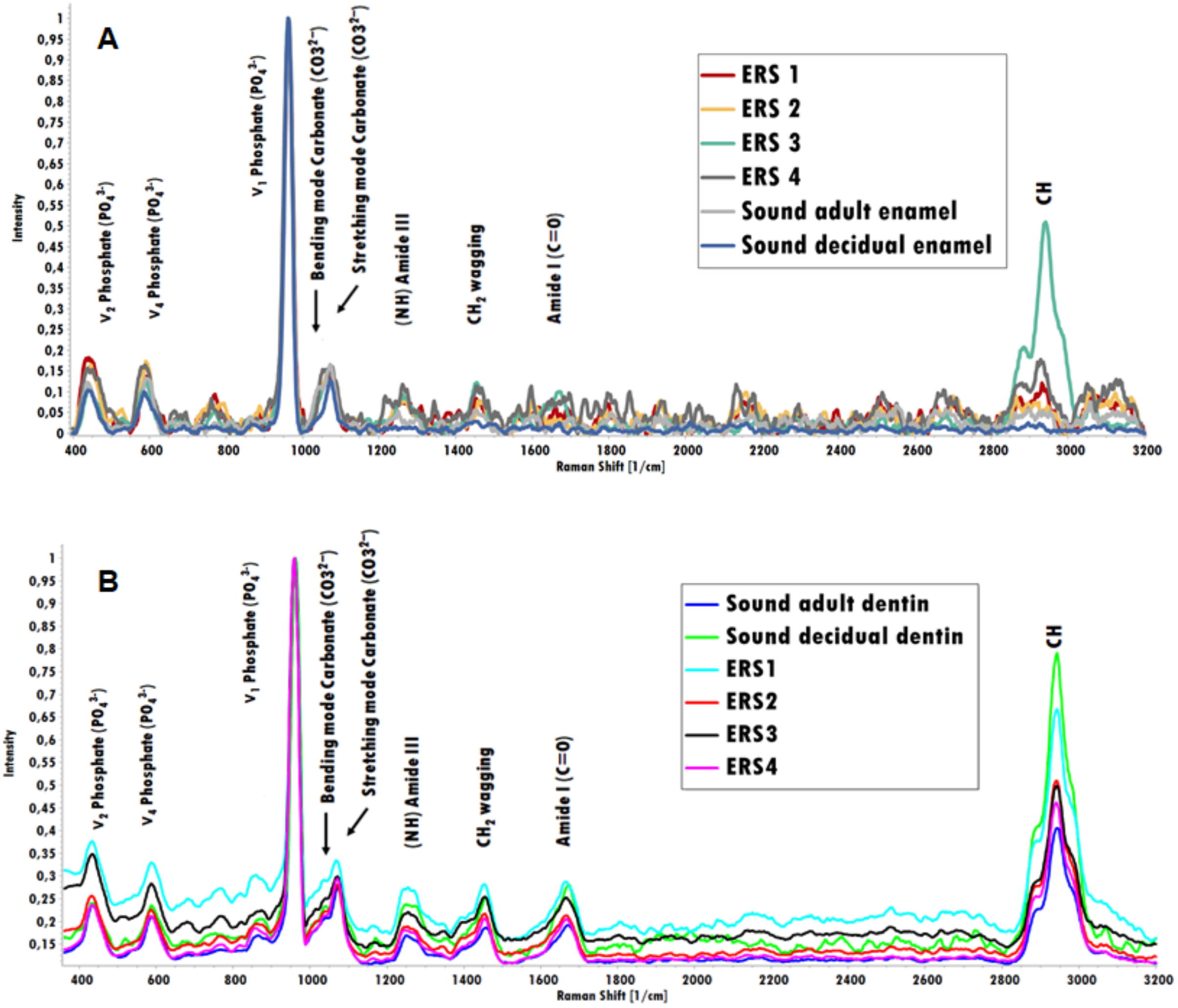

Supplemental Figure 2: normalized Raman spectra of sound (permanent and deciduous) and ERS enamel (B) and dentin (B). ERS1: c.358C>T, permanent; ERS2: c.1513delA, deciduous; ERS3: c.907_908delAG, deciduous; ERS4: c.1432C>T, deciduous

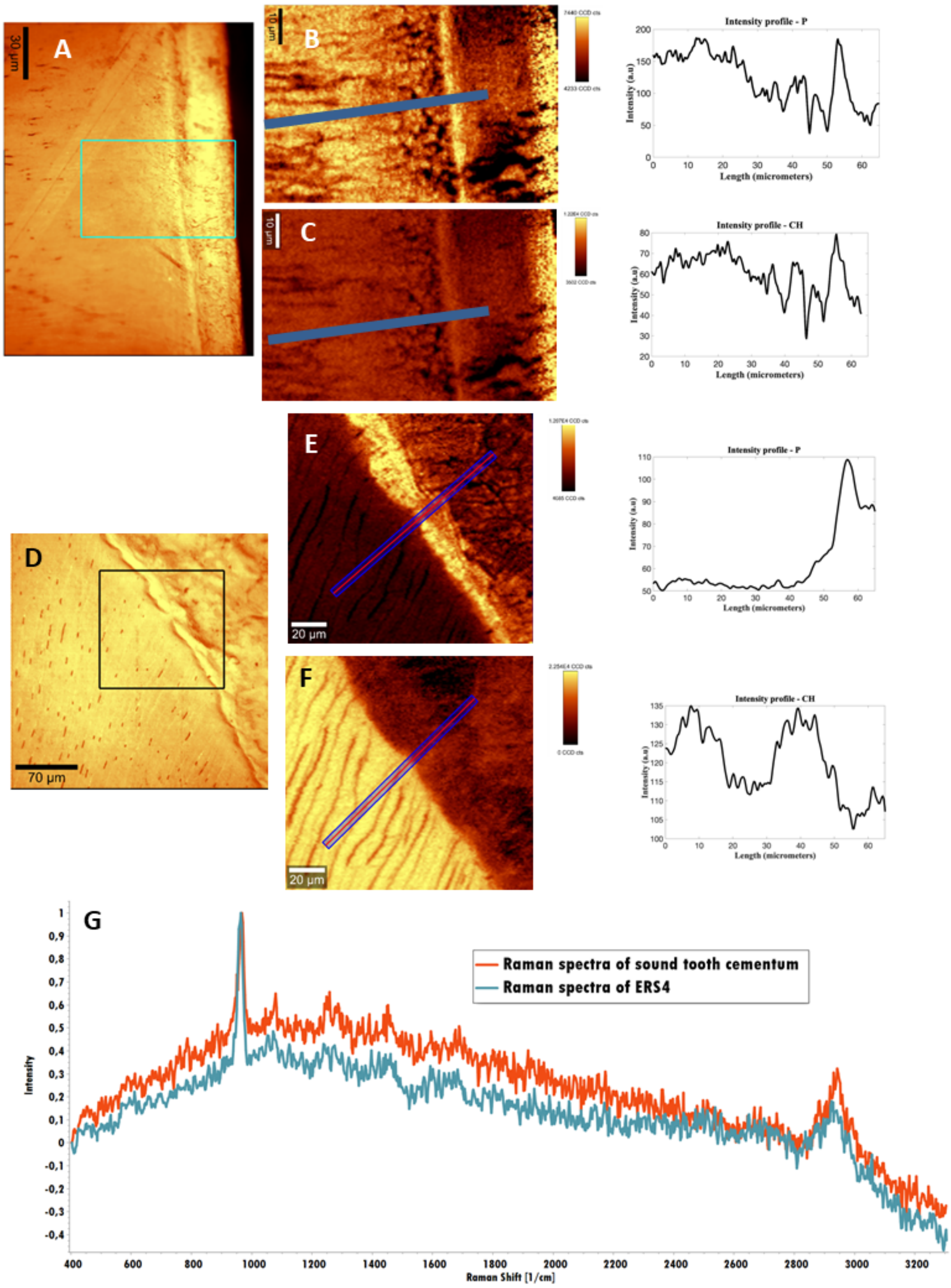

Supplemental Figure 3. Example of permanent teeth cementum scanned with confocal Raman microscopy (A-B-C) compare with ERS4 (D-E-F). A and D: x20 optical image; B and E: 960cm^{-1}phosphate peak intensity reconstructed image; C and F: 2800cm^{-1} CH peak intensity reconstructed image; next to Raman image: look up table and plotted profile of intensity,

linked with blue line. G: superposed non background substracted Raman normalized spectra of cementum of sound tooth and pseudo enamel found in ERS4. Spectra representative of the two samples.

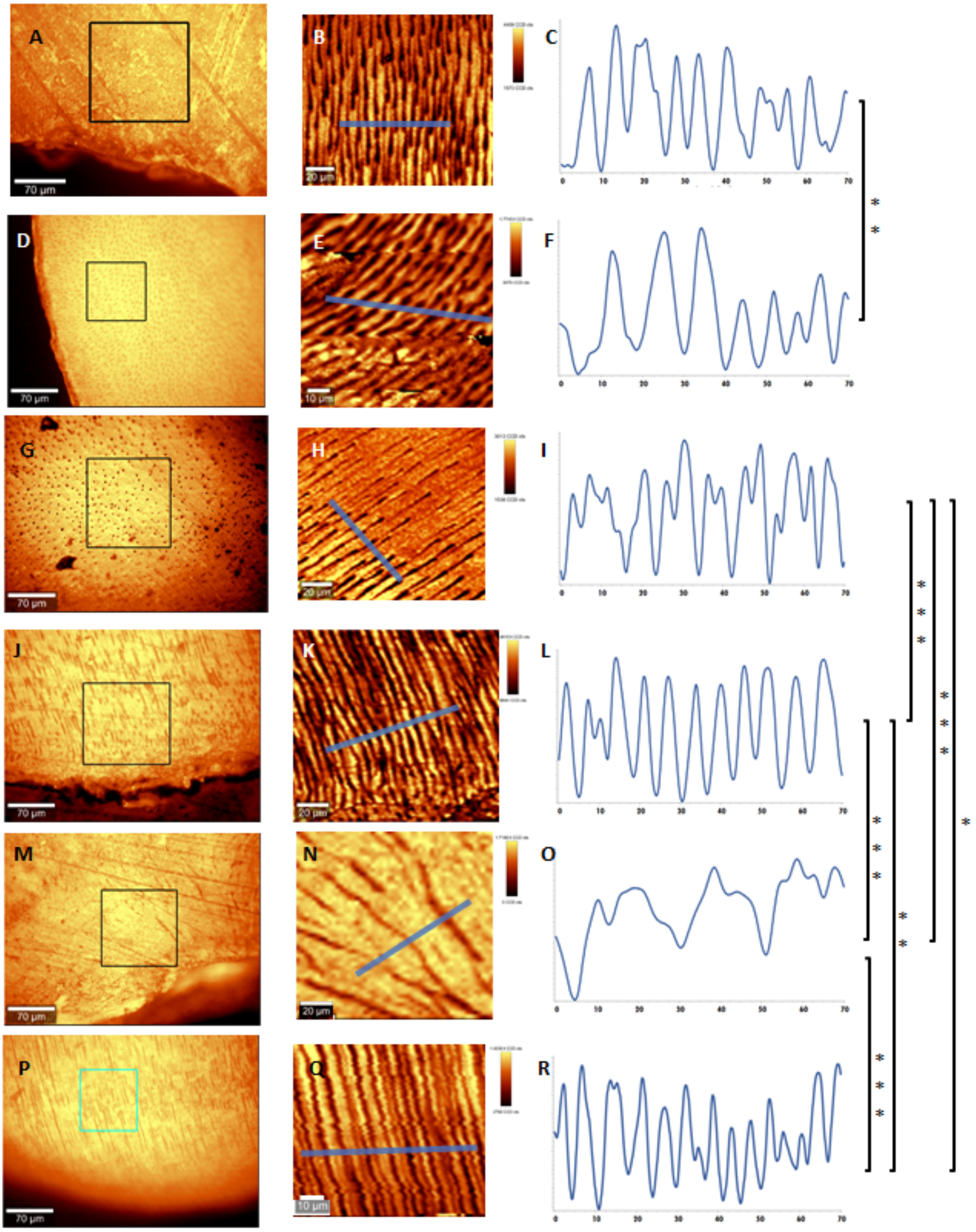

Supplemental Figure 4. Dentin structure of ERS teeth scanned with confocal Raman microscope. Left: x20 optical image. Middle: 2800cm^{-1}/CH peak intensity image. Right: 70 µm intensity profile along blue line on CH peak intensity image. A-C) sound adult dentin; D-

F) ERS1 ; G-I) sound deciduous dentin; J-L) ERS2; M-O) ERS3 ; P-R) ERS4 ; Statistical differences in dentinal tubule density are shown (*): $p<0.05$; (**): $p<0.01$; (***): $p<0.001$.

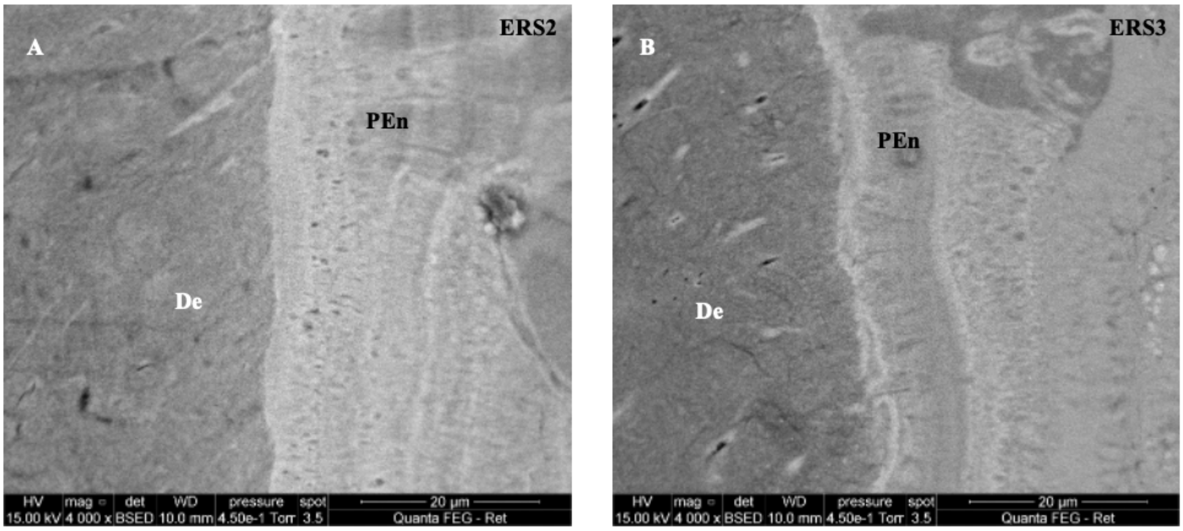

Supplemental Figure 5. Scanning electron microscope image of dentin - pseudo enamel interface. A) ERS2 ; B) ERS3. De: dentine; PEn: pseudo enamel;

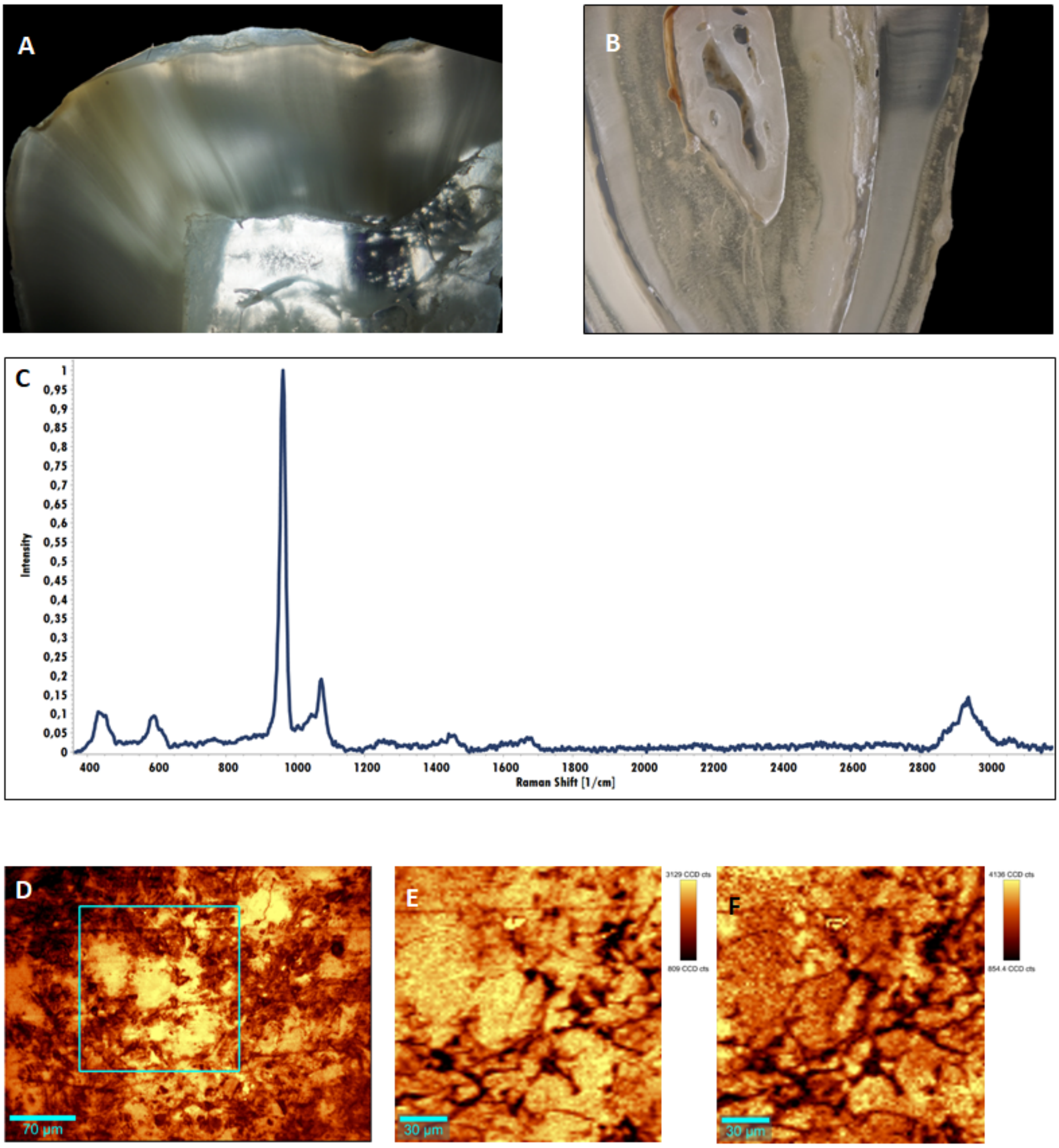

Supplemental figure 6. A and B: classical microscopy image of ERS2 crown and root, 2.5x magnification; C) single spectra of material in the pulp chamber seen on A and B with 2 seconds integration time; D) optical 20x magnification pulp chamber of ERS2; E) 960 cm^{-1} photosphate peak intensity image of the zone boxed in D; F) 2800cm^{-1} CH peak intensity image of the zone boxed in D

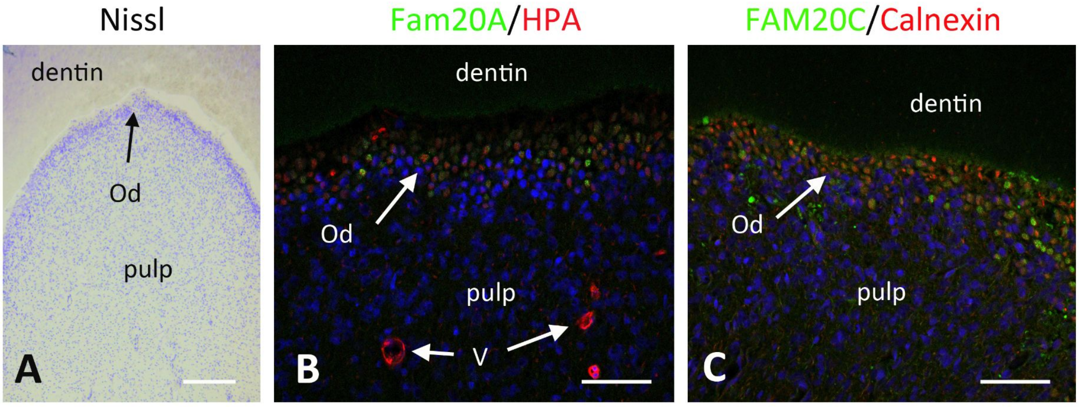

Supplemental Figure 7. FAM20A and FAM20C immunostaining. (A) Low power magnification of a section at the level of the coronary pulp stained with the vital marker, Nissl. Arrow indicated the odontoblast layer (Od). Low power magnifications indicating Fam20A (B) or FAM20C (C) expression within odontoblast layer (Od). V: vessel. Scale bars: A = 250 µm; B-C = 50 µm.

Raman band assignments	Enamel	Dentin
	(Raman shift in cm^{-1})	
ν_2 Phosphate (PO_4^{3-})	433-449	432-449
ν_4 Phosphate (PO_4^{3-})	579-608	580-610
ν_1 Phosphate (PO_4^{3-})	960	960
Bending mode Carbonate (CO_3^{2-})<br>Stretching mode Carbonate (CO_3^{2-})	1044<br>1070	1048<br>1074
(NH) Amide III		1243
(NH) Amide III non-polar triple helix of collagen		1275
CH_2 wagging		1450
Advanced glycation end products (AGEs)-Pentosidine		1550
Amide I (C=O)		1660
CH		2800-3000
OH	3580	3580
Phosphate to organic matrix ratios	960/1450	960/1450

Supplemental Table 1. Characteristic Raman peaks of enamel and dentin

	Normalized peaks intensity										
Raman shift peak (cm^{-1})	430	582	960	1045	1073	1268	1452	1666	2883	2942	2983
	ν_2 Phosph ate (PO_4^{3-})	ν_4 Phosphate (PO_4^{3-})	ν_1 Phosphate (PO_4^{3-})	Bending mode Carbonate (CO_3^{2-})	Stretching mode Carbonate (CO_3^{2-})	(NH) Amide III non-polar triple helix of collagen	CH_2 wagging	Amide I (C=O)	CH	CH	CH
ERS1-dej	0.14	0.13	1.00	0.12	0.18	0.11	0.11	0.12	0.16	0.36	0.21
ERS1-pulp	0.17	0.15	1.00	0.11	0.19	0.14	0.15	0.13	0.11	0.27	0.17
ERS4-dej	0.11	0.10	1.00	0.10	0.19	0.07	0.09	0.10	0.14	0.34	0.34
ERS4-pulp	0.13	0.11	1.00	0.11	0.21	0.12	0.16	0.18	0.26	0.62	0.30
ERS2-dej	0.13	0.13	1.00	0.13	0.18	0.11	0.13	0.14	0.19	0.47	0.27
ERS2-pulp	0.16	0.13	1.00	0.12	0.18	0.11	0.13	0.14	0.18	0.42	0.21
ERS3-dej	0.15	0.15	1.00	0.12	0.17	0.11	0.12	0.13	0.15	0.37	0.22
ERS3-pulp	-	-	-	-	-	-	-	-	-	-	-
Sound adult tooth-dej	0.16	0.18	1.00	0.17	0.22	0.18	0.19	0.19	0.26	0.51	0.31
Sound adult tooth-pulp	0.18	0.19	1.00	0.17	0.23	0.17	0.19	0.17	0.24	0.36	0.23
Sound deciduous tooth-dej	0.14	0.12	1.00	0.12	0.20	0.13	0.15	0.16	0.28	0.63	0.36
Sound deciduous tooth-pulp	0. 19	0.19	1.00	0.12	0.23	0.20	0.22	0.22	0.31	0.64	0.38

Supplemental Table 2 – normalized peak intensity; dej = line scan in dentin close to DEJ; pulp = line scan in dentin close to pulp chamber; spectra were normalized with Phosphate (PO_4^{3-})
