# Supplementary material for: Enamel and dentin in Enamel renal syndrome: A confocal Raman microscopy view
Source: Front Physiol. 2022 Aug 25;13:957110. doi: 10.3389/fphys.2022.957110 (PMC9453029; doi:10.3389/fphys.2022.957110)
Supplement: Supplementary file 3 [file Table2.docx]

|  | **Normalized peaks intensity** | | | | | | | | | | |
| --- | --- | --- | --- | --- | --- | --- | --- | --- | --- | --- | --- |
| Raman shift peak (cm^-1^) | **430** | **582** | **960** | **1045** | **1073** | **1268** | **1452** | **1666** | **2883** | **2942** | **2983** |
|  | **ν_2_ Phosphate (PO_4_^3-^)** | **ν_4_ Phosphate (PO_4_^3-^)** | **ν_1_ Phosphate (PO_4_^3-^)** | **Bending mode Carbonate (CO_3_^2−^)** | **Stretching mode Carbonate (CO_3_^2−^)** | **(NH) Amide III non-polar triple helix of collagen** | **CH_2_ wagging** | **Amide I (C=O)** | **CH** | **CH** | **CH** |
| ERS1-dej | 0.14 | 0.13 | 1.00 | 0.12 | 0.18 | 0.11 | 0.11 | 0.12 | 0.16 | 0.36 | 0.21 |
| ERS1-pulp | 0.17 | 0.15 | 1.00 | 0.11 | 0.19 | 0.14 | 0.15 | 0.13 | 0.11 | 0.27 | 0.17 |
| ERS4-dej | 0.11 | 0.10 | 1.00 | 0.10 | 0.19 | 0.07 | 0.09 | 0.10 | 0.14 | 0.34 | 0.34 |
| ERS4-pulp | 0.13 | 0.11 | 1.00 | 0.11 | 0.21 | 0.12 | 0.16 | 0.18 | 0.26 | 0.62 | 0.30 |
| ERS2-dej | 0.13 | 0.13 | 1.00 | 0.13 | 0.18 | 0.11 | 0.13 | 0.14 | 0.19 | 0.47 | 0.27 |
| ERS2-pulp | 0.16 | 0.13 | 1.00 | 0.12 | 0.18 | 0.11 | 0.13 | 0.14 | 0.18 | 0.42 | 0.21 |
| ERS3-dej | 0.15 | 0.15 | 1.00 | 0.12 | 0.17 | 0.11 | 0.12 | 0.13 | 0.15 | 0.37 | 0.22 |
| ERS3-pulp | - | - | - | - | - | - | - | - | - | - | - |
| Sound Enamel-dej | 0.16 | 0.18 | 1.00 | 0.17 | 0.22 | 0.18 | 0.19 | 0.19 | 0.26 | 0.51 | 0.31 |
| Sound Enamel-pulp | 0.18 | 0.19 | 1.00 | 0.17 | 0.23 | 0.17 | 0.19 | 0.17 | 0.24 | 0.36 | 0.23 |

**Supplementary table 2** – normalized peak intensity; dej = line scan in dentin close to DEJ; pulp = line scan in dentin close to pulp chamber; spectra were normalized with Phosphate (PO_4_^3-^)
